# Maternal adult attachment and mother-adolescent attachment: the chain mediating role of marital satisfaction and harsh parenting

**DOI:** 10.3389/fpsyt.2023.1170137

**Published:** 2023-06-20

**Authors:** Mengge Li, Huoliang Gong, Huiying Zhang, Yuping Chen, Chenze Zhang

**Affiliations:** ^1^School of Psychology, South China Normal University, Guangzhou, Guangdong Province, China; ^2^Institute of Psychology and Behavior, Henan University, Kaifeng, Henan Province, China; ^3^School of Business, Henan University, Kaifeng, Henan Province, China

**Keywords:** maternal adult attachment, marital satisfaction, harsh parenting, mother-adolescent attachment, adolescence

## Abstract

This study explored the relationship between maternal adult attachment and mother-adolescent attachment based on the attachment theory and spillover hypothesis of family systems theory. A survey research was conducted on 992 mothers and adolescents using a convenience sampling method. A survey research was conducted on 992 Chinese mothers and adolescents using a convenience sampling method. The results indicated that (1) maternal adult attachment avoidance and anxiety were significantly negatively related to maternal marital satisfaction and mother-adolescent attachment, and significantly positively related to maternal harsh parenting; maternal adult attachment anxiety was a significant direct predictor of mother-adolescent attachment; (2) Maternal marital satisfaction and maternal harsh parenting mediated the significant effect between maternal adult attachment and mother-adolescent attachment, specifically pathways contained three: a separate mediating effect of maternal marital satisfaction, a separate mediating effect of maternal harsh parenting, and a chain mediating effect of maternal marital satisfaction and maternal harsh parenting. The findings suggest that maternal adult attachment, marital satisfaction, and harsh parenting behaviors can have significant effects on adolescents’ mother-adolescent attachment.

## 1. Introduction

The child-rearing philosophy of Chinese parents emphasizes the responsibility of parents for their children’s behavior. The traditional belief in “spare the rod, spoil the child” has been deeply respected, leading to more educational guidance and assistance provided to children. However, in order to cultivate good behavior in their children, parents may also resort to harsh parenting such as physical punishment (1). In Chinese society and culture, the mother remains the primary caregiver in most families, and thus has more interaction with the child. This may also result in more frequent use of harsh disciplinary practices in order to foster good behavior in the child (2).

In recent years, there have been cases of mothers in China scolding and kicking their teenagers for being playful or failing to meet academic standards, causing them to run away from home or commit suicide (3). What motivates mothers to verbally abuse and kick their children? How does the attachment relationship between mothers and children become so incompatible that children choose to run away from their mothers rather than give up their lives? Adolescence is a critical period in the shaping of attachment as individuals move from childhood to adulthood (Cassidy and Shaver, 2002) (4). Adolescents’ growing sense of independence and autonomy, but their desire to rely on their mothers for troubles as they did as children, and the coexistence and conflict between independence and dependence all make mother–child attachment in adolescence complex and difficult to grasp (5) (Steinberg & Morris, 2001) (6). The quality of mother-adolescent attachment has important implications for adolescents’ psychosocial adjustment (7), psychological quality (8), depression (9), and other mental health problems (10). As mothers are the primary caregivers of adolescents, exploring the formation mechanisms of mother-adolescent attachment can help reduce mothers’ harsh parenting style, improve the quality of mother-adolescent attachment, and promote adolescents’ mental health.

According to the internal working model of attachment, mothers’ adult attachment not only affects the pattern of interaction between mothers and husbands, but also affects the pattern of interaction with adolescents, which in turn affects the quality of attachment between adolescents and mothers (11), but it is unclear exactly how maternal adult attachment avoidance and attachment anxiety affect the mechanism of action of mother-adolescent attachment. According to the spillover hypothesis of family systems theory, there are marital subsystems, mother–child subsystems, and father-child subsystems in families, and the marital subsystem affects the parent–child subsystem (12); therefore, mothers’ harsh parenting behaviors are likely to be influenced by mothers’ marital satisfaction. There is a strong relationship between maternal marital satisfaction and maternal adult attachment, and the ability of mother-adolescent attachment to reflect maternal marital satisfaction to some extent has been found by numerous researchers (Mikulincer and Shaver, 2008) (13–15). Thus, the quality of mother-adolescent attachment in adolescents is likely to be influenced by maternal adult attachment, marital satisfaction, and maternal harsh parenting, but the exact mechanisms of action are unclear. In order to investigate the formation mechanism of mother-adolescent attachment, this study used mothers and adolescents as research subjects to examine the effects of maternal adult attachment, marital satisfaction, and harsh parenting on the quality of mother-adolescent attachment in adolescents, and to provide a new research perspective for improving the quality of mother-adolescent attachment and promoting family harmony.

### 1.1. Maternal adult attachment and mother-adolescent attachment quality

Hazan and Shaver (16) extended attachment theory to the adult stage, arguing that the emotional connection to a partner in a romantic relationship can also be viewed as an attachment relationship, i.e., adult attachment. Brennan et al. (17) proposed a dimensional view of attachment, dividing attachment into two sequential dimensions: attachment anxiety and attachment avoidance, with individuals who are attachment avoidant preferring to distance themselves from their partners and avoid their partners’ intimate individuals with adult attachment anxiety fear that they will be abandoned by their partners, desire intimacy, and have more controlling behaviors toward their partners. According to the internal working model of attachment (11), adult attachment avoidant mothers develop positive perceptions of self and negative perceptions of others and feel fearful of avoiding the intimate needs of others to avoid being hurt by the intimate relationship. Individuals with adult attachment anxiety develop an internalized negative self-perception and positive other-perception, resulting in individuals with adult attachment anxiety often believing that they are not good enough and always fearing abandonment in their intimate relationships. Such cognitive and interactional patterns may be present not only in couple interactions but also in interactions with children. Mothers who are adult attachment avoidant may respond to their adolescents’ needs by not responding and avoiding them, and mothers who are adult attachment anxious may overreact to their children’s needs and thus develop more controlling behaviors toward their children (18). Poor interaction patterns in both adult attachment avoidant and anxious mothers are more likely to prevent good quality of mother-adolescent attachment between adolescents and their mothers. Therefore, this study proposes research hypothesis 1: maternal adult attachment avoidance and anxiety negatively predict mother-adolescent attachment quality.

### 1.2. The mediating role of marital satisfaction

Maternal marital satisfaction refers to the degree of satisfaction that a mother has with her own marriage (19). Sandber et al. (20) showed that individuals with either adult attachment avoidance or adult attachment anxiety were negatively associated with their marital satisfaction. Mothers who are adult attachment avoidant are more likely to exhibit inactive response patterns such as avoidance and indifference in the marital relationship, and are more independent and fearful of wanting to avoid their partner’s intimacy needs, etc., which can lead to a decrease in marital satisfaction for adult attachment avoidant mothers in the marital relationship (21). Adult attachment-anxious mothers are more likely to use over-activation strategies to deal with intimacy and repeatedly confirm that their husbands love them, which can make their husbands feel untrusted and uncontrolled and may engage in more escapist behaviors from their mothers, which further increases the anxiety of attachment-anxious mothers and thus decreases marital satisfaction (22).

Mother-adolescent attachment refers to the stable, lasting, and deep emotional bond formed between adolescents and their mothers (23). According to family systems theory, family systems are composed of different subsystems: couple subsystem, parent–child subsystem (mother–child subsystem, father-child subsystem), etc., and the different subsystems interact with each other. Adult attachment belongs to the couple subsystem in the family system, and mother-adolescent attachment belongs to the mother–child subsystem (12). Regarding the influence of the couple subsystem on the mother-son subsystem, the spillover hypothesis of family systems theory suggests that emotions or behaviors that arise in one subsystem (e.g., the couple subsystem) are expressed in another subsystem (e.g., the mother–child subsystem) (12). That is, if mothers’ marital satisfaction is low, it indicates that mothers are likely to transfer their own unpleasant emotional experiences and inappropriate behaviors in the marital relationship to their interactions with their children, thereby reducing the quality of mother-adolescent attachment. It has been shown that mothers’ marital satisfaction is strongly correlated with the quality of attachment between mothers and adolescent (24). Therefore, this study proposes hypothesis 2: maternal marital satisfaction is likely to mediate the relationship between mothers’ adult attachment and mother-adolescent attachment quality.

### 1.3. The mediating role of mother’s harsh parenting

Harsh parenting is mainly manifested by parents’ physical (e.g., slapping, spanking) and verbal (e.g., verbal abuse, yelling) aggressive behaviors toward children, as well as harsh emotions and attitudes such as boredom, indifference, anger, moodiness, and insensitivity (25) (Wang et al., 2016) (26). Both adult attachment avoidant and anxious mothers are always unresponsive and inappropriate when responding to adolescents’ need for support (27), adult attachment avoidant mothers are more likely to respond to adolescents’ negative emotions with unacceptance, anger, etc. (28), and adult attachment anxious mothers are more likely to respond to adolescents’ mothers with adult attachment anxiety are more likely to react erratically or excessively to their adolescents’ negative emotions (e.g., scolding or intimidation) (29). Harsh parenting behaviors by mothers can disrupt the safe, warm and comfortable interaction patterns between mothers and adolescents, making the interaction between adolescents and mothers unpleasant and oppressive, and the adolescents’ increasing sense of autonomy and harsh parenting behaviors can lead to a lot of dissatisfaction and rebellion toward mothers, which inevitably deteriorates the quality of mother-adolescent attachment (30). Therefore, this study proposes hypothesis 3: maternal harsh parenting mediates the relationship between maternal adult attachment and mother-adolescent attachment.

### 1.4. The chain mediating role of maternal marital satisfaction and maternal harsh parenting

Mothers’ lower marital relationship satisfaction implies poor marital quality, and mothers generate more dysphoria during poor couple interactions, which spills over into the mother–child subsystem and affects the mother-adolescent interaction process (12), and mothers with dysphoria interact with their adolescents with a lack of patience, abandoning planned, rational, and child-centered discipline strategies and rely on passive, easy-to-implement, and mother-centered strategies such as harsh parenting behaviors (31, 32). It has been shown that low maternal marital satisfaction leads mothers to interact with adolescents through insensitive, irritable, and other abusive behaviors, and this pattern of interaction leads to defiance and hostility toward the mother, which in turn exacerbates the mother’s abusive parenting strategies, creating a vicious cycle in which the interaction process inevitably worsens the quality of mother-adolescent attachment (33). Mothers’ adult attachment quality can reflect mothers’ marital satisfaction (34). Therefore, this study proposes hypothesis 4: Maternal marital satisfaction and harsh parenting play a chain mediating role between maternal adult attachment and mother-adolescent attachment.

**Figure 1:**
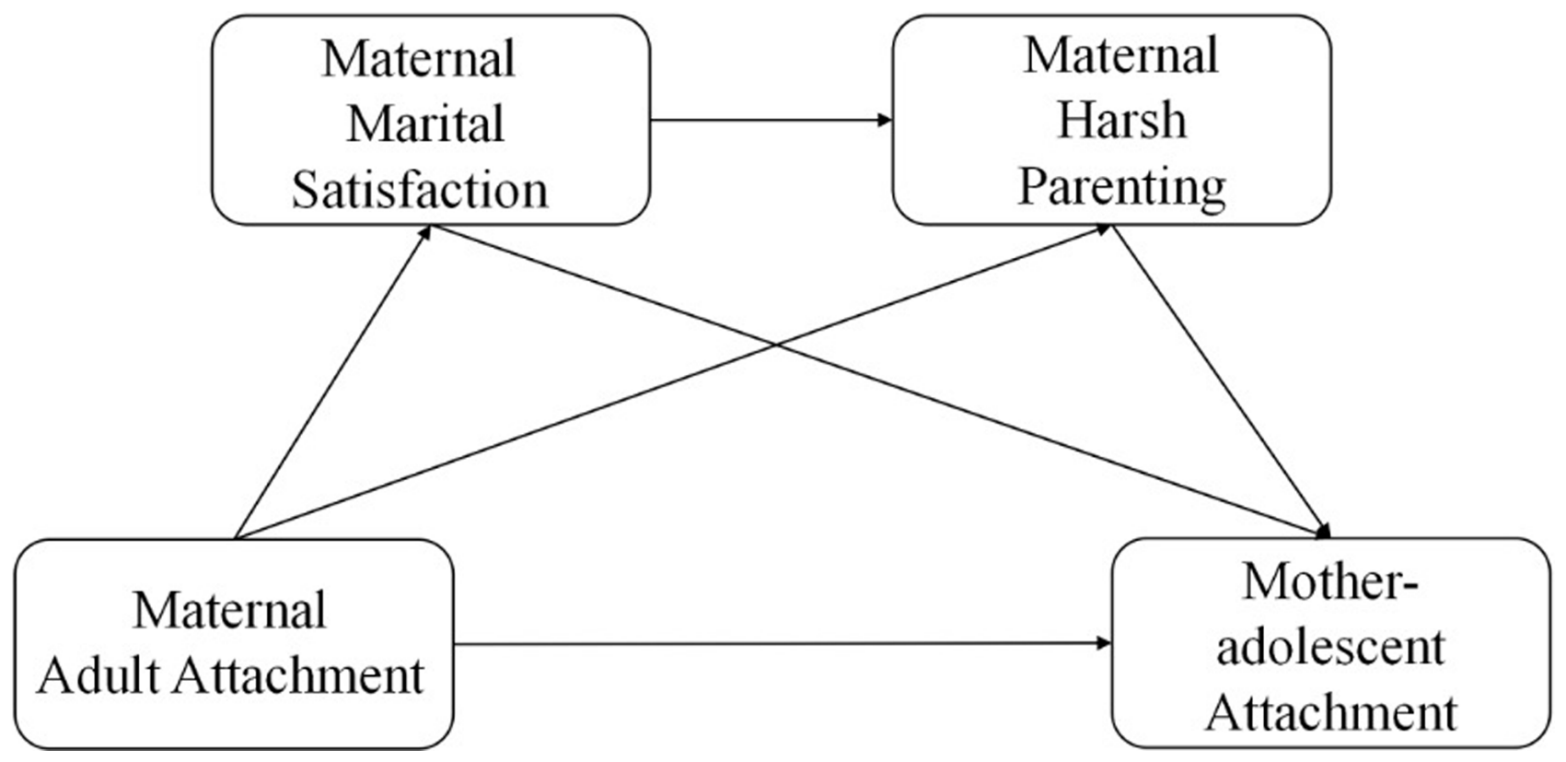
Hypothetical model diagram.

Based on attachment theory and family systems theory, this study, for the first time, examines the relationship between mothers’ adult attachment and mother-adolescent attachment, as well as the mediating role of mothers’ marital satisfaction and mothers’ harsh parenting, to provide more perspectives for improving the quality of mother-adolescent attachment and enhancing family harmony and well-being, and attempts to construct the following model (Figure 1).

## 2. Methods

### 2.1. Participants

A total of 992 pairs of mothers and adolescents participated in this study using a convenience sampling method to measure some adolescents and their mothers in China. The mean age of the adolescents was 13.56 (SD = 1.20), with 51.40% of the boys and 48.60% of the girls, and the mean age of the mothers was 40.60 (SD = 5.69).

### 2.2. Procedure

Prior to data collection, the researcher introduced the study to the adolescents and the participants were informed that they could withdraw from the study at any time and that the test would be administered only with the consent of the mother and adolescent. Adolescents independently completed the questionnaire in the classroom. After completing the questionnaire, the adolescents brought it back home to be filled out by their mothers. Once the mothers completed the questionnaire, the adolescents brought it back to school. The study was approved by the ethics committee of the authors’ institution. In this study, data with more than three unanswered items or with obvious patterns indicating that all items were selected or a particular option was consistently chosen, were excluded from analysis.

### 2.3. Measures

#### 2.3.1. Mother-reported adult attachment

The Experiences in Close Relationship Scale (ECR), revised by Li and Kato (35), was used to measure maternal adult attachment scores. The scale has 36 entries, divided into two dimensions: attachment avoidance and attachment anxiety, with odd-numbered questions scoring the attachment avoidance dimension and even-numbered questions scoring the attachment anxiety dimension, using a seven-point scale. Higher scores represent higher levels of adult attachment avoidance and anxiety in mothers. In the present study, the Cronbach’s alpha coefficients for the attachment avoidance and attachment anxiety scales for mothers were 0.81 and 0.91, respectively.

In this study, the results of confirmatory factor analysis for adult attachment with mother met the standard requirements: *χ^2^/df* = 4.022, CFI = 0.968, TLI = 0.951, SRMR = 0.059, and RMSEA = 0.055 [0.050, 0.061]. The confirmatory factor analysis for adult attachment anxiety with mother met the standard requirements: *χ^2^/df* = 4.942, CFI = 0.955, TLI = 0.934, SRMR = 0.053, and RMSEA = 0.063 [0.058, 0.069].

#### 2.3.2. Mother-reported marital satisfaction

The Marital Satisfaction Questionnaire (MSQ) is a subquestionnaire of the Olson Marital Quality Questionnaire (EN-RICH) (36) with 10 items. five-point scale, with 1 being “really so” and 5 being “really not so.” The higher the score, the higher the marital satisfaction of the mother. The internal consistency coefficient of mothers’ marital satisfaction was 0.79, respectively. In this study, the results of confirmatory factor analysis for marital satisfaction with mother met the standard requirements: *χ^2^/df* = 2.676, CFI = 0.990，TLI = 0.982, SRMR = 0.018, and RMSEA = 0.041 [0.029, 0.053].

#### 2.3.3. Mother-reported harsh parenting

The parental harsh parenting questionnaire developed by Wang (37) was used to measure fathers’ harsh parenting. For example, “When my child does something wrong or makes me angry, I lose my temper and even yell at my child.” The questionnaire is a five-point scale that mothers are asked to complete. The higher the score, the more frequent the mother’s harsh parenting behavior. The internal consistency coefficient of mothers’ harsh parenting was 0.89, respectively. In this study, the results of confirmatory factor analysis for harsh parenting met the standard requirements: *χ^2^/df* = 1.295, CFI = 1.000，TLI = 0.999，SRMR = 0.004，RMSEA = 0.017 [0.000, 0.068].

#### 2.3.4. Adolescent-reported mother-adolescent attachment

The mother-adolescent attachment subquestionnaire of the Inventory of Parent and Peer Attachment (IPPA), developed by Armsden and Greenberg (5) and revised by Li et al. (38), was used to measure the mother-adolescent attachment subquestionnaire, which was completed by adolescents. The 13-item questionnaire was divided into three dimensions: trust, communication, and estrangement. Trust represents the level of trust that the child has in their mother, communication represents the quality of communication between the child and their mother, and estrangement represents the psychological distance between the child and their mother. A five-point scale was used, and the higher the score, the higher the quality of mother-adolescent attachment between the adolescents and their mothers. The Cronbach’s alpha coefficient of the mother-adolescent attachment scale in this study was 0.85. In this study, the results of confirmatory factor analysis for mother-adolescent attachment met the standard requirements: *χ^2^/df* = 4.580, CFI = 0.971，TLI = 0.963, SRMR = 0.035, and RMSEA = 0.060 [0.053, 0.067].

### 2.4. Data analysis

First, the data were analyzed for reliability, descriptive statistics, and correlation analysis using SPSS 22.0, and second, the chain mediated effect test was performed using the Amos software version 24. The indirect effect was the bias correction of the sample distribution using a 95% confidence interval with 5,000 repetitions.

## 3. Results

### 3.1. Descriptive statistics and correlation analysis

SPSS 22.0 was used to conduct normality analysis on the data. The results showed that the skewness of maternal adult attachment avoidance was |−0.29|<2.0, with kurtosis of |−0.44|<7.0; the skewness of maternal adult attachment anxiety was |0.47|<2.0, with kurtosis of |0.45|<7.0; the skewness of maternal marital satisfaction was |0.34|<2.0, with kurtosis of |−0.50|<7.0; the skewness of maternal harsh parenting was |0.94|<2.0, with kurtosis of |0.68|<7.0; and the skewness of mother–child attachment was |−0.21|<2.0, with kurtosis of |0.23|<7.0. According to previous research, data with |skewness|<2.0 and |kurtosis|<7.0 are approximately normal (39), indicating that the data for maternal adult attachment avoidance, maternal adult attachment anxiety, maternal marital satisfaction, maternal harsh parenting, and mother–child attachment are all approximately normal.

Descriptive statistics and correlation analyses were performed for each variable, and the results are shown in Table 1.

**Table 1:** Mean, standard deviation, and correlation coefficient of each variable (*N = 992*).

	*M*	*SD*	1	2	3	4	5	6	7	8
1. Age	-	-	1							
2. Gender	-	-	−0.03	1						
3. AHI	-	-	−0.12	0.01	1					
4. MAA1	3.17	0.88	−0.01	−0.02	0.01	1				
5. MAA2	3.40	1.16	0.07	−0.05	−0.04	0.44^**^	1			
6. MMS	3.64	0.73	−0.01	−0.01	0.01	−0.68^**^	−0.32^**^	1		
7. MHP	2.00	0.94	0.04	−0.03	−0.01	0.33^**^	0.44^**^	−0.40^**^	1	
8. MCA	3.84	0.72	0.01	0.02	−0.01	−0.22^**^	−0.22^**^	0.27^**^	−0.26^**^	1

AHI, annual household income; MAA1, maternal adult attachment avoidance; MAA2, maternal adult attachment anxiety; MMS, maternal marital satisfaction; MHP, maternal rough parenting; and MCA, maternal child attachment; ^**^p < 0.01.

As shown in Table 1, there is no significant correlation between gender, age and annual family income and mother-adolescent attachment; mother’s adult attachment avoidance is positively correlated with mother’s adult attachment anxiety, mother’s harsh parenting, and negatively correlated with mother’s marital satisfaction and mother-adolescent attachment; mother’s adult attachment anxiety is positively correlated with mother’s harsh parenting, and negatively correlated with mother’s marital satisfaction and mother-adolescent attachment. Maternal attachment anxiety was negatively related to maternal harsh parenting, maternal satisfaction, and mother-adolescent attachment; maternal harsh parenting was negatively related to maternal satisfaction and mother-adolescent attachment; maternal harsh parenting was negatively related to mother-adolescent attachment.

### 3.2. Chain mediating effect test

Amos version 24 was used to conduct a chain mediation analysis on the data, and the correlation analysis showed that there were no significant correlations between adolescents’ age, gender, and annual family income and adolescents’ mother–child attachment, so they were not used as control variables. Using maternal adult attachment avoidance as the independent variable, mother-adolescent attachment as the dependent variable, and maternal marital satisfaction and maternal harsh parenting as mediating variables, a chain mediated effect analysis with 5,000 replicate sampling distributions was conducted using the Amos version 24. Due to the fact that all three variables in this model are single-dimensional and the number of items is relatively small, a manifest variable structural equation model was constructed. The model index showed a df = 0, because in this case the covariance matrix has 10 elements (5 × 4 × 0.5), and there are six path coefficients and four variables’ variances to be estimated, for a total of 10 parameters. The degrees of freedom is 10–10 = 0, indicating that it is a saturated model. Therefore, the model is saturated and all indicators are optimal.

**Figure 2:**
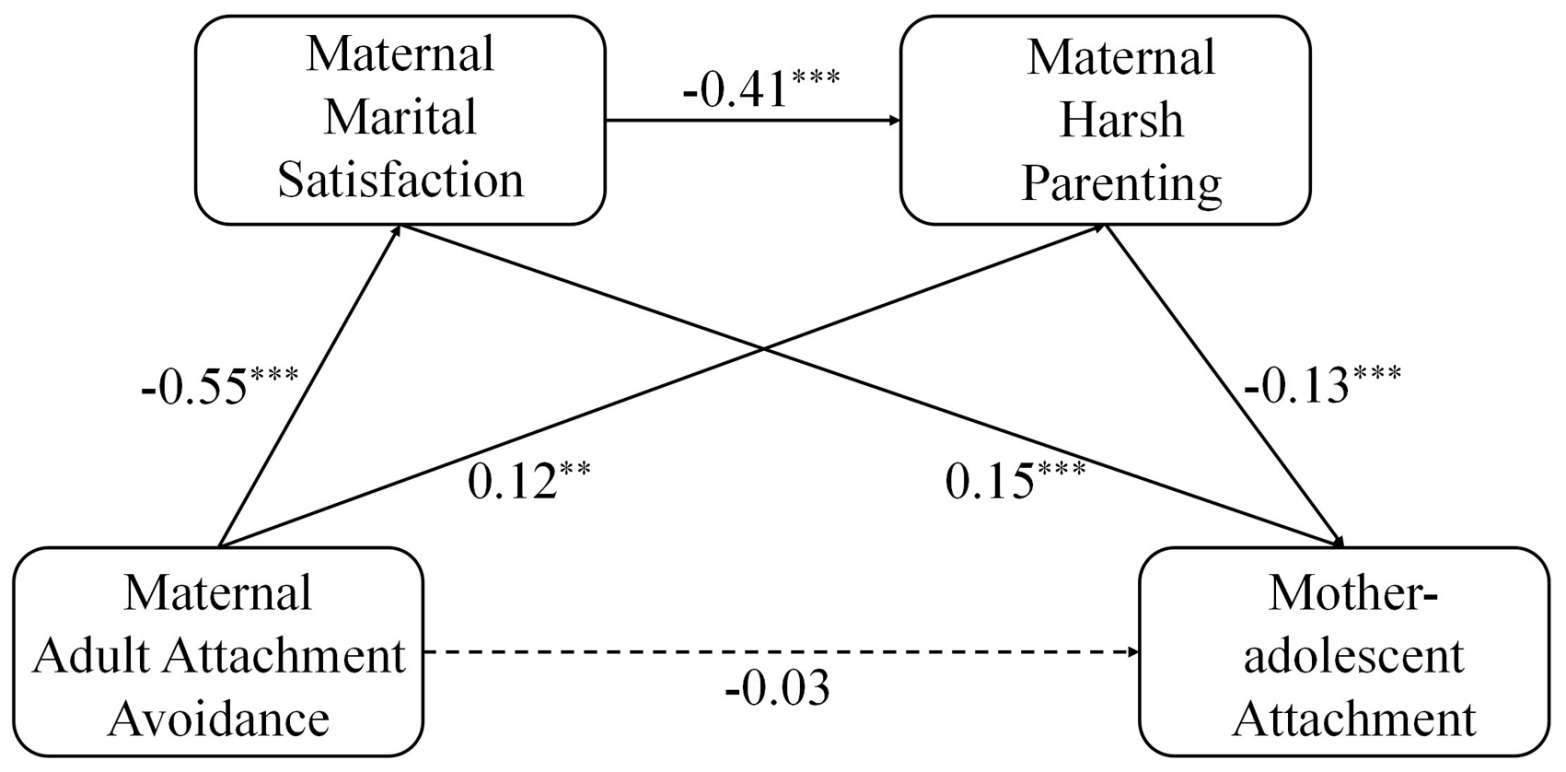
The chain meditation diagram of mother’s adult attachment avoidance and mother-adolescent attachment. ^**^p < 0.01, ^***^p < 0.001.

The results are shown in Figure 2. Maternal adult attachment avoidance positively predicted maternal marital satisfaction (β = −0.55, *p* < 0.001). Maternal adult attachment avoidance positively predicted maternal harsh parenting behavior (β = 0.12, *p* < 0.01). Maternal marital satisfaction negatively predicted maternal harsh parenting behavior (β = −0.41, *p* < 0.001). Maternal adult attachment avoidance was not a significant direct predictor of mother-adolescent attachment quality (β = −0.03, *p* > 0.05). Maternal marital satisfaction significantly positively predicted mother-adolescent attachment quality (β = 0.15, *p* < 0.001). Maternal harsh parenting negatively predicted adolescent mother-adolescent attachment quality (β = −0.13, *p* < 0.001). Bootstrap analysis revealed a significant mediating effect of maternal marital satisfaction between maternal adult attachment avoidance and mother-adolescent attachment (indirect effect = −0.09, *p* < 0.01), a significant mediating effect of maternal harsh parenting between maternal adult attachment avoidance and mother-adolescent attachment (indirect effect = −0.02, *p* < 0.01), and a significant chain effect of maternal marital satisfaction and maternal harsh parenting between maternal adult attachment avoidance and mother-adolescent attachment (indirect effect = −0.03, *p* < 0.001).

**Figure 3:**
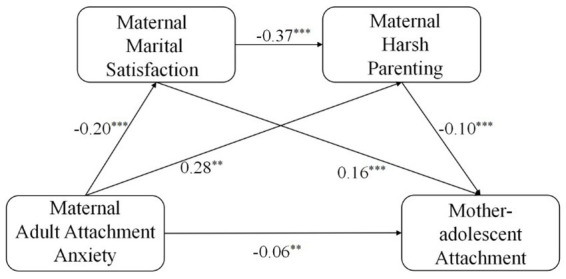
Chain meditation diagram of maternal adult attachment anxiety and mother-adolescent attachment. ^**^p < 0.01, ^***^p < 0.001.

Using mothers’ adult attachment anxiety as the independent variable, mother-adolescent attachment as the dependent variable, and mothers’ marital satisfaction and mothers’ harsh parenting as mediating variables, the results of the analysis with 5,000 replicate sampling distributions were conducted using the Amos version 24 as shown in Figure 3.

Maternal adult attachment anxiety positively predicted maternal marital satisfaction (β = −0.20, *p* < 0.001). Maternal adult attachment anxiety positively predicted maternal harsh parenting behavior (β = 0.28, *p* < 0.001). Maternal marital satisfaction negatively predicted maternal harsh parenting behavior (β = −0.37, *p* < 0.001). Maternal adult attachment anxiety was a negative predictor of mother-adolescent attachment (β = −0.06, *p* < 0.01). Maternal marital satisfaction positively predicted mother-adolescent attachment quality (β = 0.16, *p* < 0.001). Maternal harsh parenting negatively predicted mother-adolescent attachment quality (β = −0.10, *p* < 0.001). Bootstrap analysis revealed a significant mediating effect of maternal marital satisfaction between maternal adult attachment anxiety and mother-adolescent attachment (indirect effect = −0.03, *p* < 0.001), a significant mediating effect of maternal harsh parenting between maternal adult attachment anxiety and mother-adolescent attachment (indirect effect = −0.03, *p* < 0.001). The chain mediating effect between maternal marital satisfaction and maternal harsh parenting was significant between maternal adult attachment anxiety and mother-adolescent attachment in adolescents (indirect effect = −0.01, *p* < 0.001).

## 4. Discussion

### 4.1. Maternal adult attachment and mother-adolescent attachment

The results suggest that maternal adult attachment anxiety directly predicts mother-adolescent attachment, which is consistent with some of the existing research findings (7). Mothers with adult attachment anxiety adopt an over-activation strategy in their dealings with their adolescents, and this strategy leads to more control and emotional instability during the mother-adolescent interaction. Mothers with adult attachment anxiety are prone to develop an internal working model of whether the other person thinks I am bad, a model that causes her to repeatedly confirm whether she is doing a good job and whether she will be recognized and loved by the adolescent, to be oversensitive to signals that threaten the relationship and to overreact to maintain the relationship (40), but adolescence is a critical period of increased self-awareness (41), this pattern of being together can cause adolescents to constantly want to escape from their mothers’ excessive validation and control, and adolescents’ avoidance makes mothers want to further strengthen their control, which leads to a decrease in the quality of adolescent-mother attachment (7). Adult attachment avoidant mothers do not directly predict adolescents’ mother–child attachment quality, inconsistent with some of the existing research findings (42). As adolescents are in the transition from home to school, adolescents have more support from teachers and peers (43), and this support is likely to buffer the stress and strain on adolescents caused by adult attachment-avoidant mothers’ unresponsiveness to adolescents, and would not directly contribute to lower adolescent-mother attachment quality.

### 4.2. Mediating role of maternal marital satisfaction

The results suggest that maternal marital satisfaction mediates the relationship between maternal adult attachment and mother-adolescent attachment, consistent with some previous research (33, 44). Both adult attachment-avoidant and anxious mothers adopt more anachronistic patterns of interaction in their marital relationships causing marital tension (attachment-avoidant mothers adopt more avoidant, nonresponsive ways of responding to their partners’ intimacy needs, and attachment-anxious mothers over-control their partners and repeatedly confirm their worthiness of being loved, both of which strategies contribute to marital tension and thus reduce marital satisfaction) (45). According to the spillover hypothesis of family systems theory, mothers’ emotions and behaviors in the marital subsystem spillover to the parent–child subsystem to influence mother–child interaction processes, which in turn affects the quality of mother-adolescent attachment (12).

### 4.3. The mediating role of maternal harsh parenting

The results suggest that maternal harsh parenting mediates the relationship between maternal adult attachment and mother-adolescent attachment, which is consistent with the study hypothesis. Mothers with adult attachment avoidance and adult attachment anxiety were less able to regulate and control their own emotions than mothers with secure adult attachment and faced more difficulties in regulating their own emotions (46). Mothers who are attached to avoidance and anxiety in their marital life have more dysphoria and conflict with their husbands than mothers who are attached to security (47), and this dysphoria is transferred to interactions with adolescents. Adolescents are in a critical period of increasing independence and autonomy (41), disagreeing with their mothers on more things and mothers are faced with more conflict situations with their adolescents. Adolescents are more likely to take a relatively simple and violent approach to conflict resolution, which undoubtedly hinders the expression of adolescent autonomy and can damage the quality of the mother attachment (48).

### 4.4. Chain mediating role of marital satisfaction and harsh parenting

The findings suggest that maternal marital satisfaction and harsh parenting play a chain mediating role between maternal adult attachment and mother-adolescent attachment, consistent with the hypothesis of this study. Mothers with adult attachment avoidance and anxiety are more likely to have poor couple interaction patterns with their partners and are more likely to have adverse emotional experiences in the marital relationship, which can reduce mothers’ marital satisfaction (20). Adolescence is a critical period of increasing independence (41), as adolescents no longer follow their mothers’ advice and there are more disagreements between mothers and adolescents. Mothers value their children’s schooling, and if children are playful or fail to do well in school, this can create disagreements between mothers and adolescents. Adverse emotions generated by mothers during poor couple interactions can affect the quality of mother-adolescent interactions, and in these negative emotional states, mothers are more likely to respond to their adolescents with anger and hostility when they disagree with them, and are more likely to respond to their adolescents’ disobedience with aggressive verbal abuse and other harsh parenting behaviors (49). This also validates the family systems theory spillover hypothesis that mothers who are dissatisfied with their marital relationship are more likely to have more dysphoria in their marital relationship, and this dysphoria makes mothers more likely to adopt self-centered, violent, and other repressive tactics in their interactions with their adolescents, which in turn deteriorates the quality of mother-adolescent attachment (12, 30, 48). Mothers should reflect on whether they are doing so because of a bad marital relationship or the adolescent is really behaving inappropriately before preparing to rough up their children, which will help reduce mothers’ poor parenting behaviors toward their adolescents due to a bad marital relationship and thus improve the quality of mother-adolescent relationships.

## 5. Research implications

This study investigates the formation path of mother-adolescent attachment based on attachment theory and family systems theory, which has certain theoretical and practical significance for improving the quality of mother-adolescent attachment: (1) This study uses mothers and adolescents as subjects to examine how adult attachment of mothers within families affects adolescents’ mother-adolescent attachment, which is useful for further research on how adult attachment of parents affects parenting behavior and parent-adolescent attachment in the future. It has some implications for further research on how parental adult attachment affects parenting behavior and parent-adolescent attachment. (2) This study has some implications for the use of family systems theory therapy to intervene in family relationships. Although both maternal adult attachment avoidance and anxiety can adversely affect mother-adolescent attachment, maternal adult attachment anxiety can directly affect the quality of mother-adolescent attachment, which inspires us that interventions for maternal adult attachment anxiety in family therapy are more important for family harmony. This inspires us that when conducting family therapy, interventions for mothers’ adult attachment anxiety are more important for family harmony and improvement of mother-adolescent relationships. (3) Family therapists can also use training programs, such as positive thinking training to reduce harsh parenting (50), to enhance the emotional regulation of mothers with adult attachment avoidance and anxiety in the face of parenting stress, increase more appropriate parenting behaviors, and promote the healthy development of parent-adolescent attachment.

## 6. Limitations and future directions

Although this study has some practical significance for improving the quality of mother-adolescent attachment among adolescents, there are still the following limitations, which need further improvement in the future. (1) First, this study used a cross-sectional design, and the findings could not reveal the causal relationship between variables, for example, mother’s harsh parenting may also affect mother’s marital satisfaction, and future studies could use a follow-up design to examine the influence mechanisms of maternal adult attachment, marital satisfaction, harsh parenting, and mother-adolescent attachment. (2) In the family system, not only the mother–child system but also the father-child system has an important impact on adolescent attachment. This study only explored the factors influencing mother-adolescent attachment from the perspective of maternal adult attachment and did not investigate the pathway of father-adolescent attachment formation. Adolescence is also a key period for the development and change of father-adolescent attachment, and future research can further explore the impact of paternal adult attachment on father-adolescent attachment. (3) The subjects of this study were collected only in central China and not in the more economically developed coastal cities, which have lower incomes compared to coastal cities, so the results of this study have limitations for extending to adolescent families with better economic conditions, and future studies should involve a broader study population to enhance the scope of extension of the study results.

## 7. Conclusion

This study explored the effects of maternal adult attachment, marital satisfaction, and harsh parenting on mother-adolescent attachment based on attachment theory and family systems theory. The results indicate that maternal adult attachment has a direct effect on mother-adolescent attachment, and marital satisfaction and harsh parenting play a chain-mediating role between maternal adult attachment and mother-adolescent attachment.

## Data availability statement

The raw data supporting the conclusions of this article will be made available by the authors, without undue reservation.

## Ethics statement

The studies involving human participants were reviewed and approved by Ethical Review of Psychological Research in Henan Province Key Laboratory of Psychology and Behavior. Written informed consent to participate in this study was provided by the participants’ legal guardian/next of kin.

## Author contributions

ML: conceptualization, methodology, software, investigation, and writing-original draft preparation. HG: conceptualization, methodology, writing guidance, and writing-review and editing. HZ: software and analysis or interpretation of data. YC: make writing suggestions. CZ: formatting and proofreading. All authors contributed to the article and approved the submitted version.

## Funding

This study was supported by study on the influence and intervention of academic Autobiographical memory on adolescent learning adjustment—2021 Henan Provincial Philosophy and Social Science Planning Annual Project (project number: 2021BJY004).

## Conflict of interest

The authors declare that the research was conducted in the absence of any commercial or financial relationships that could be construed as a potential conflict of interest.

## Publisher’s note

All claims expressed in this article are solely those of the authors and do not necessarily represent those of their affiliated organizations, or those of the publisher, the editors and the reviewers. Any product that may be evaluated in this article, or claim that may be made by its manufacturer, is not guaranteed or endorsed by the publisher.
